# Community-Based Pulmonary Rehabilitation in an Economically Deprived Area of Jodhpur, India: A Mixed-Methods Feasibility Trial

**DOI:** 10.2147/COPD.S488766

**Published:** 2025-02-28

**Authors:** Mahendra Thakor, Vishal Singh, James Manifield, Mark W Orme, Pankaj Bhardwaj, Nishant Kumar Chauhan, Amy C Barradell, Zahira Ahmed, Yashika Bhati, Jesse Matheson, Andy Barton, Arun Kumar Sharma, Sally J Singh

**Affiliations:** 1 ICMR-National Institute for Implementation Research on Non-Communicable Diseases, Jodhpur, India; 2 Centre for Exercise and Rehabilitation Science, Department of Respiratory Sciences, University of Leicester, Leicester, UK; 3 Centre for Exercise and Rehabilitation Science, NIHR Leicester Biomedical Research Centre-Respiratory, University Hospitals of Leicester, Leicester, UK; 4 All India Institute of Medical Sciences, Jodhpur, India; 5 Department of Economics, University of Sheffield, Sheffield, UK; 6 Department of Community Medicine, University College of Medical Sciences, New Delhi, India

## Introduction

Pulmonary rehabilitation (PR) has been shown to be a highly effective intervention for people living with COPD, with improvements observed in exercise capacity, health-related quality of life, and dyspnoea. In low-to-middle-income countries (LMICs), such as India, the need for PR greatly exceeds capacity. Individuals with COPD from economically deprived areas of India are at a significant disadvantage due to the underutilisation of PR (ie, being limited to the private sector)^1^ and the high associated healthcare costs.

Current evidence in India has shown that dependence on caregivers for travel to a rehabilitation facility, the strain of healthcare expenses on family, low self-esteem of the participants, and lack of awareness or recognition of the PR benefits hinders individuals from participating in rehabilitation programmes.^2^ Various modes of PR delivery, such community-based PR in non-healthcare facilities, have been utilised to combat these barriers in both high-income countries (HICs) and LMICs and have been shown to be safe, viable, and efficacious for those unable to access PR in a specialised facility.^3^

Accordingly, the aim of this study was to investigate the feasibility and acceptability of community-based PR for individuals with COPD residing in economically deprived areas of Jodhpur, Rajasthan, India.

## Materials and Methods

Participants were recruited onto a single-arm trial (ISRCTN10069208 and CTRI/2021/11/037877) approved by Ethical Review Committee of the ICMR-NIIRNCD (IEC-ICMR-NIIRNCD/2021/25/11), AIIMS Jodhpur (AIIMS/IEC/2021/3740) and University of Leicester (32024) as part of the National Institute for Health and Care Research (NIHR) Global Health Research group on Pulmonary Rehabilitation (Global RECHARGE) project.^4^ The trial was carried out at Radhe Krishna community hall near to an economically deprived (Rajiv colony) area of Jodhpur, Rajasthan. All participants provided written informed consent prior to study commencement.

Inclusion criteria were aged 18–65 years (in accordance with safety guidelines during the COVID-19 pandemic) with confirmed diagnosis of COPD, able to give informed consent, ≥1 exacerbation in the last 12 months, and a Medical Research Council (MRC) dyspnoea grade of ≥2. Patients with co-morbidities precluding exercise were excluded. A door-to-door survey of approximately 800 households was planned to identify individuals suffering from respiratory issues in the last six months and subsequently recruit 40 individuals with COPD. People reporting significant respiratory symptoms and had not undergone a spirometry test in last 12 months were invited for spirometry, conducted as per ATS/ERS guidelines.

The community-based PR intervention was developed and conducted in accordance with the British Thoracic Society’s quality standards and comprised of 12 face-to-face sessions, delivered over 6 weeks (2 sessions/week with a minimum of 2 days between sessions). These sessions were conducted at a community centre located approximately 1.5 km away from the Rajiv colony. Each session lasted for 2 hours (1 hour supervised exercise [both endurance and strength training] and 1 hour group education). Exercises were individually prescribed, and tailored for low-resource setting, ie, by incorporating low cost and self-made equipment such as water bottles instead of dumbbells. Participants were also encouraged to perform daily exercises at home and were provided with exercise diaries tailored to the literacy level of included participants.

The PR programme was led by a physiotherapist and assisted by two staff nurses who were trained by health professionals with expertise in PR from University Hospitals of Leicester NHS Trust, UK. Meal vouchers were provided to participants after each session as an incentive. Transport was provided to participants within a 2km radius.

Feasibility outcomes were (i) recruitment (percentage of eligible patients who were recruited) and (ii) completion (defined as attending ≥75% sessions [≥9/12 sessions] and attending follow-up assessment). These outcomes were based on a traffic light system where green indicated the feasibility of trial using the set methodology, amber indicated the need of modifications in the methodology and red indicated non-feasibility of the trial. Separate predetermined thresholds were used for (i) recruitment (green ≥60%; amber 25–59%; red <25%) and (ii) completion (green ≥70%; amber 50–69%; red <50%). Irrespective of recruitment rate, data collection had to be completed by the end of March 2023 due to project restraints, giving 1 year to recruit the target sample size. Termination of the trial would occur if this was not achieved.

The acceptability of PR among individuals with COPD (PR completers and non-completers) and HCWs was assessed via face-to-face semi-structured interviews conducted by a female Master of Public Health student (YB), who was fluent in the local language (Marwari) and not involved in PR delivery. Main topics included the experience of the PR, perceived benefits, challenges and barriers, and any suggestions for future programmes. Interviews were conducted in Hindi and Marwari languages and audio recorded, transcribed to Hindi (when applicable) and further translated to English to support thematic analysis.

Quantitative data were calculated as frequencies, percentages, medians, and interquartile ranges within IBM SPSS Statistics (version. 28). Thematic analysis, employing a codebook approach, was used to generate themes from interview data.

## Results

**Figure 1:**
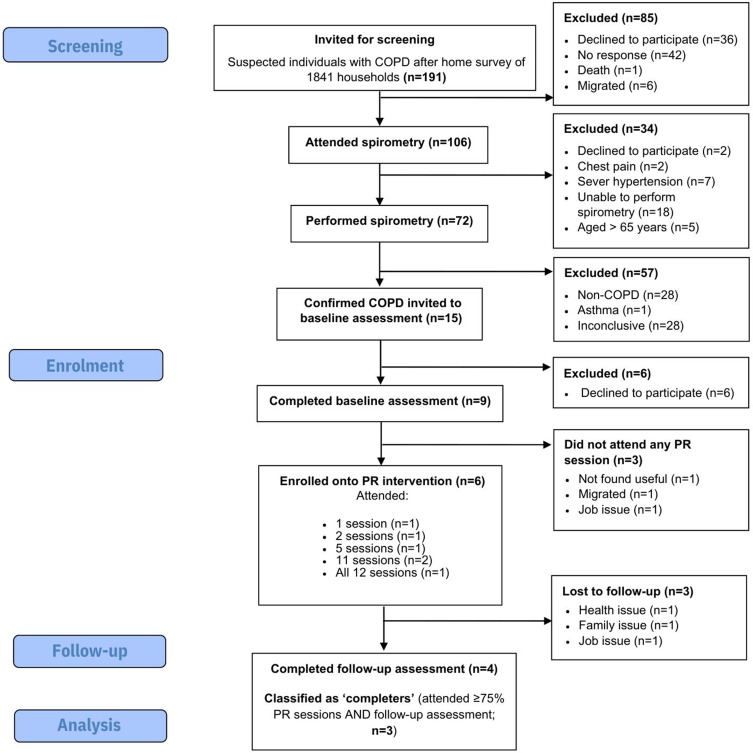
CONSORT flow diagram.

1841 households were approached, and 191 suspected individuals were invited for spirometry, of which, 106 (55%) attended the assessment (Figure 1). Fifteen (19%) had confirmed COPD (all newly diagnosed) and invited for the baseline assessment. Of these, nine (60% of eligible) attended and were recruited to the study (median [IQR] age: 56 [53–60], 88.9% male, 66.7% illiterate). In accordance with our stopping guidelines, the study was terminated on the 31st March 2023 following the recruitment of 9/40 participants. Three (33% of recruited) completed at least 75% of sessions and attended the post-intervention assessment, three participants were lost to follow-up, and the remaining three were considered non-completers (Figure 1).

Interviews were conducted with 4 hCWs (2 nurses, 1 physiotherapist, 1 project coordinator) and 7 patients (3 completers, 4 non-completers). Four themes were generated. Illustrative quotes are provided in Table 1.

**Table 1:** Challenges Identified During the Study and Proposed Solutions for Future Research

Challenges and Illustrative Quotes	Proposed Solutions
**Lack of awareness towards COPD and PR** My work is such that I naturally do exercise. Because I do labour work, so it is exercise for me. (J00009)	Earlier implementation of education during screening/recruitment process for both patients and family members
**Limited advocacy for PR from clinicians and participants** According to me, if their doctor, who is already treating them, tells them that they should tell to do exercise and also prescribe medicines for them, then maybe they will accept his advice more easily. (HCW1)	Implement targeted training for clinicians on the benefits of PR to increase referrals and patient advocacy Utilise patient testimonials and experiences to promote the programme and encourage participation
**Low PR attendance due to conflicts with work** I did not get leave [to attend PR sessions] and if I took leave, they used to deduct the money. (J00001) My knowledge is that morning time, before 2 pm, before lunch, this period is good… Get free by 9:00, make your husband’s or any child’s meal, get free, then join here. (J00007)	Schedule classes outside standard working hours (eg, early mornings, evenings) to accommodate working participants
**Lack of family support** Some of them had a family issue, that means the family members used to refuse. Because of that, we faced some problems due to job, and some had family issues. (HCW4) Most of the females had the problem that their husbands would not have allowed them, that’s why those females would not have been able to come. (HCW1)	Encourage family members to attend PR sessions (especially education sessions) to improve knowledge and awareness
**Low levels of literacy in this population** I am an illiterate person, you are educated, so they used to talk after looking at the paper, I do not know how to read, but I used to understand it. (J00003)	Offer extra support during PR sessions via 1-to-1 conversations to ensure participants understand all information provided to them
**Travel to PR venue** Those who did not have a vehicle, they would come on foot, that’s why their blood pressure would increase, their breath would become short, so even then the baseline assessment could not be done. (HCW3)	PR venue located in densely populated area to minimise travel costs and/or move to a more home-based approach
**Preference for pharmacological treatments over PR** One patient used to say that if our doctor has not asked us to do exercise and he just write medicines for us and we are getting instant relief from it, so why should we do exercise? (HCW1)	Increase PR awareness of prescribing clinicians
**Seasonal factors (ie, low temperature (6–14°C) in winter) preventing attendance** There were some dropouts in the PR programme who could not continue due to weather conditions. Breathing difficulties are more common during winters. Our PR programme was conducted in winter, around December-January, and at that time, they faced difficulties. So, they could not participate. (HCW4)	Introduce flexibility in programme structure to accommodate seasonal variations and patient needs

Theme 1: Unmet COPD health needs show the need for community-based PR: This study involved participants being diagnosed with COPD during the screening process, allowing for the identification of individuals who were previously unaware of their condition, highlighting the impact of COPD on their daily functioning and leading to a better understanding of their respiratory issues. Patients described the disease’s impact on their daily functioning and reflected on the physical limitations which often go unrecognized as part of their healthcare management.

Theme 2: The central role of HCWs delivering community-based PR: The HCWs described establishing a patient-centred and safety-driven programme specifically tailored for these individuals with illiteracy (eg, simple exercises, interactive educational sessions in the local language, and providing a friendly environment). The patients reported trusting the HCWs who delivered the PR and felt as though the exercises and education components were relevant to their COPD condition.

Theme 3: Facilitators and Barriers to community-based PR: Very few facilitators were reported in the present study. Those that were in the catchment area for the provision of transport support described this as a major facilitator to attendance. Furthermore, the provision of meal vouchers was also viewed positively. The main challenges to PR recruitment and completion, with respective quotes and proposed solutions, are reported in Table 1. In brief, these included lack of COPD and/or PR awareness (resulting in fear and resistance amongst the community due to the association of breathing problems with COVID-19), and lack of family support, as some family members would not allow the patients to participate. Daily wage workers reported needing to sacrifice a day of paid work to attend which caused financial concern. Participants were hesitant to perform spirometry due to the stigma associated with the test. Some patients perceived the exercises to be unnecessary, as they felt they were already engaging in physically demanding housework. HCWs also recounted the social and cultural barriers faced by female participants in the PR programme due to gender-based restrictions within this community.

Theme 4: Perceived benefits of participating in community-based PR: Participants reported improvements such as enhanced functional capacity, physical and psychological relief from breathing difficulties. HCWs believed this to signify the progressive benefits of PR. The outcomes were not solely limited to physical benefits but were also evident in patients’ overall improvements in well-being and quality of life.

Our findings suggest that our community-based PR programme was not feasible in its current format. Recruitment was terminated early due to challenges during the recruitment procedures; however, those engaging with the programme appeared to benefit. We have identified considerations and improvements needed to facilitate future trials of PR in this disadvantaged and underrepresented population (Table 1).

## Discussion

A lack of health literacy, including knowledge about COPD as a disease or spirometry as a diagnostic test, may have contributed to the reluctance to complete the survey, spirometry assessment, and/or participate in the study.^5^ This is a particularly important barrier as our screening process involved newly diagnosing COPD. Furthermore, the timing of introducing PR immediately after a COPD diagnosis may have left some patients feeling overwhelmed, impacting their willingness to engage in the programme.

The low literacy level observed in individuals from this economically deprived area likely affected uptake rate at various levels of the screening process. Difficulty was experienced when performing spirometry assessments due to poor compliance and technique from individuals, leading to many inconclusive results.

The lack of knowledge of PR and its benefits was evidenced by some of our participants perceiving exercise to be unnecessary due to viewing their manual jobs as their exercise, and their preference to pharmacological treatments due to the immediate symptomatic relief. In Pune, India, a recent qualitative study^2^ support our finding regarding the lack of awareness to PR as an important barrier, resulting in patients not addressing long-term strategies to manage their COPD. This has also been observed by Bickton and Shannon^6^ in other LMICs, who suggested that the bias towards medications over rehabilitation may be due to the ratio of low number of rehabilitation professionals to number of medical doctors in these areas.

Another major barrier to PR in this study concerned job-related issues, specifically the loss of wages when attending PR, which has been previously reported in other LMICs.^6^ This is of particular importance in the context of economically deprived areas as the majority of our participants had a monthly household income of <6400 rupees/month (~$77). In HICs, participants living in more deprived areas are less likely to complete PR. The reported lack of family support may have contributed to the low uptake of PR in this population and setting. Previous research has highlighted the importance of family-involvement with PR programmes.^7^ Furthermore, the lack of women recruited in the current study was likely due to cultural views and restrictions within the population. In a study investigating the perceptions of PR in Pune, India,^2^ women often reported that household work restricts their ability to adhere to exercise routines - a barrier that was not observed in men.

The use of a multi-methods approach to evaluate the feasibility and acceptability of the community-based PR programme provided us with a holistic understanding of the challenges and potential solutions which can be applied to future research. Adapting the PR programme to suit a low-resource environment, using self-made and low-maintenance equipment, underscores our commitment to making the intervention relevant to participants in an economically deprived area of India. Our door-to-door survey approach and reliance on self-reported respiratory symptoms for initial screening could have introduced selection bias. The upper age limit of 65 years was implemented in accordance with COVID-19 safety guidelines at the time to minimise associated risk and obtain ethical approval. As only ~3% of screened individuals in this study were older than 65 years, it is unlikely that the inclusion of these individuals would have changed the trial findings. The lack of a control group limits our ability to attribute observed changes solely to the intervention. While meal vouchers might improve short-term adherence, there is limited insight into long-term adherence and maintenance of exercise routines post-intervention.

## Conclusion

In conclusion, whilst the present study found that our community-based PR in an area of low socio-economic status in India was not feasible, it does offer an opportunity for future research to build on the lessons learned when implementing PR in this underrepresented population of India.
